# Nanocellulose-Reinforced Poly(Lactic Acid) and Poly(ε-caprolactone) Bio-Nanocomposites: A Review and Future Outlook for Poly(Lactic Acid)/Poly(ε-caprolactone) Blend Systems

**DOI:** 10.3390/ma18225172

**Published:** 2025-11-14

**Authors:** Mbongeni Ngwenya, Thandi Patricia Gumede, Ricardo Arpad Pérez Camargo, Bennie Motloung

**Affiliations:** 1 Department of Life Sciences, Central University of Technology, Free State, Bloemfontein 9301, South Africa; mbongeningwenya96@gmail.com; 2 POLYMAT and Department of Polymers and Advanced Materials: Physics, Chemistry and Technology, Faculty of Chemistry, University of the Basque Country UPV/EHU, Paseo Manuel de Lardizábal, 3, 20018 San Sebastián, Spain; riky0712@hotmail.com; 3 Department of Chemistry and Polymer Science, Stellenbosch University, Private Bag X1, Matieland 7602, South Africa

**Keywords:** poly(ε-caprolactone), poly(lactic acid), nanocellulose, processing, PLA/PCL blends, nanocomposites, biodegradable, mechanical reinforcement

## Abstract

The growing demand for sustainable materials has intensified research on biodegradable polymers, particularly poly(ε-caprolactone) (PCL), poly(lactic acid) (PLA), and their blends. PLA and PCL offer biocompatibility and biodegradability, making them attractive for biomedical, packaging, and agricultural applications; however, their practical utility remains limited owing to intrinsic drawbacks. PLA has low impact strength and poor thermal resistance, while PCL suffers from low tensile strength and slow degradation kinetics. Blending PLA with PCL can complement their properties, providing a tunable balance of stiffness and flexibility. Further improvements can be achieved through the incorporation of micro- and nanocellulose (NC), which act as reinforcements, nucleating agents, as well as compatibilizers. We critically examine fabrication strategies for NC-reinforced PLA, PCL, and their blends, highlighting NC extraction, surface modification, processing strategies, and dispersion techniques that prevent agglomeration and facilitate uniform distribution. Comparative insights into composite and nanocomposite systems reveal that NC incorporation significantly enhances mechanical properties, thermal resistance, crystallization, and biodegradation kinetics, particularly at low filler loadings, owing to its high surface area, specific strength, and hydrophilicity. The review underscores the potential of PLA/PCL-based nanocomposites as eco-friendly biomaterials with tunable properties tailored for diverse sustainable applications.

## 1. Introduction

Biodegradable polymers, including poly(lactic acid) (PLA) and poly(ε-caprolactone) (PCL), have gained significant attention. On one hand, PLA is derived from renewable resources such as corn starch, which provides degradation under specific conditions. On the other hand, PCL, despite being a synthetic polymer, also offers biodegradable characteristics. These polymers offer an alternative to petroleum-based plastics and align with the increasing demand for sustainable materials. However, both PLA and PCL exhibit limitations that restrict their wide-scale use. As summarized in Table 1, PLA and PCL exhibit contrasting properties. PLA, despite its high tensile strength and rigidity, is brittle and prone to fracture [1,2], while PCL, known for its flexibility, suffers from low mechanical strength [3]. The combination of these polymers in blends aims to balance these properties, though the immiscibility of the two remains a challenge [4]. To address these limitations, different kinds of fillers, such as inorganic nanoparticles, carbon-based fillers, metal and metal oxides, bio-based nanofillers, and cellulose-based nanofillers, have been explored for the enhancement of PCL and PLA properties [5,6,7,8,9,10,11,12,13]. In particular, nanocellulose (NC) has emerged as an attractive nanofiller for these polyesters and their blends in the development of fully biodegradable nanocomposites. Beyond its inherent biodegradability, biocompatibility, and non-toxicity, NC exhibits high specific strength and tunable surface chemistry, setting it apart from inorganic, carbon-based, and metal-based fillers that may pose potential health or environmental concerns. These features, combined with their renewable origin, make it a promising and sustainable alternative for designing high-performance bio-nanocomposites. Recent research has focused on reinforcing these polymers and their blends with NC, either in the form of cellulose nanocrystals (CNCs), cellulose nanofibrils (CNFs), or bacterial nanocellulose. In PLA-based systems, NC has been shown to enhance tensile strength, Young’s modulus, and crystallinity [14,15,16,17,18,19]. This is due to its ability to act as a nucleating agent and reinforce the polymer structure at the nanoscale. For instance, studies have reported up to 40–60% improvement in tensile strength with the addition of well-dispersed CNCs at low loadings (1–5 wt.%), along with improved thermal stability [16]. Similarly, PCL-based systems exhibited increased stiffness and biodegradation rate [20,21,22], especially when surface-modified NC is used to improve interfacial adhesion with the more hydrophobic PCL matrix [23]. Beyond individual matrices with NC, research has also focused on PLA/PCL blend-based nanocomposites, where NC plays a dual role: reinforcing the polymer network [24] and acting as a compatibilizer [25,26,27,28,29,30]. Generally, in immiscible polymer blends, nanocellulose localizes at the interface between PLA and PCL phases, reducing interfacial tension and promoting stress transfer across both phases. This results in finer phase morphologies and an improvement in the mechanical synergy and dimensional stability. In some studies, the use of NC has yielded PLA/PCL blend composites with balanced toughness and strength, improved elongation at break and enhanced thermal behavior [24,27,31,32]. Moreover, techniques such as melting extrusion and blending, solvent casting, injection molding and 3D printing have been used to optimize dispersion and alignment of NC within the matrix, directly influencing composite performance [24,25,26,27,31,32,33,34]. Table 1 presents the summary of some of the PCL and PLA properties, such as tensile strength, elongation at break, melting temperature and glass transition temperature. It also presents the processing methods, approximate degradation under different composting conditions and applications of PCL and PLA.

This review provides a comprehensive overview of the synthesis and properties of PCL, PLA, and their blends, with a particular focus on the incorporation of different forms of NC as reinforcements to enhance their mechanical, thermal, and biodegradation performance. It is structured to first discuss the individual characteristics of PCL and PLA, followed by NC extraction and characterization, and then details the preparation, properties, and applications of PCL/NC, PLA/NC, and PLA/PCL blend-based bio-nanocomposites, including hybrid reinforcements.

## 2. Synthesis and Properties of Poly(ε-caprolactone)

**Figure 1:**
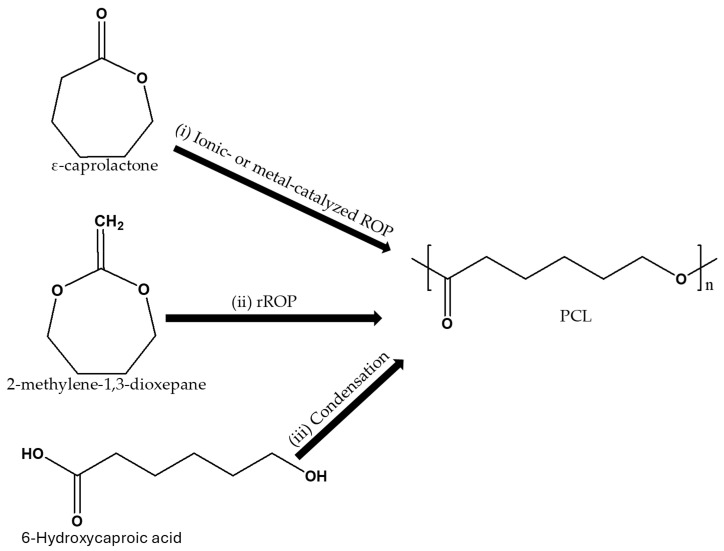
Various synthetic routes for the production of PCL (Adapted from [45], Open Access).

Poly(ε-caprolactone) (PCL) is an aliphatic polyester that is widely recognized for its biodegradability, biocompatibility, and ease of processing, making it a material of interest in both biomedical and environmental applications. According to literature, PCL is synthesized via (i) ring-opening polymerization (ROP) (ionic- or metal-catalyzed, and coordination insertion method) of the cyclic monomer ε-caprolactone, (ii) cyclic ketene acetals using radical ring-opening polymerization (rROP), and (iii) 6-hydroxycaproic acid polycondensation, as shown in Figure 1 [39]. ROP remains the most widely used method due to its high efficiency, ability to control molecular weight, and compatibility with large-scale production. Moreover, the ROP of ε-caprolactone process is highly sensitive to the choice of catalysts, which include stannous octoate, aluminum alkoxides, as well as zinc-based and tin-based complexes. The choice of a catalyst affects the molecular weight distribution of the resulting PCL, which in turn influences its mechanical and thermal properties [40]. For example, the zinc–cobalt double-metal cyanide complex [Zn-Co(III) DMCC] produced higher molecular weight PCL, which exhibits better tensile strength and elongation at break, but may compromise processability due to its increased viscosity [41]. The recent literature has increasingly focused on alternative synthesis routes, including enzyme-catalyzed and organo-catalytic polymerization, to address concerns about the residual toxicity of tin-based catalysts such as stannous octoate in biomedical applications [42,43,44]. In one study, the role of steric hindrance in the catalytic efficacy during polymerization was investigated. For this purpose, tin(IV)-based catalysts such as tetramethyltin, dimethyldiphenyltin, triphenyltin chloride and tetraphenyltin were utilized and compared. The latter was reported to be effective in promoting propagation and thus produce PCL with high molecular weight, narrow dispersity, and low oligomer content. The resulting polymer exhibited superior tensile strength and elongation at break compared to the PCL synthesized using other catalysts [44].

In terms of properties, PCL is semi-crystalline, with spherulites consisting of lamellae oriented in parallel and separated by amorphous regions with a reported degree of crystallinity of *ca*. 40%, a melting temperature in the range of 58–64 °C, and a glass transition temperature in the range of −65 to −60 °C (see Table 1) [35,46,47]. These thermal properties contribute to its high flexibility and low-temperature processability. Mechanical studies have shown that PCL typically exhibits moderate tensile strength (10–40 MPa) and remarkable elongation at break (up to 600%), depending on the molecular weight, degree of crystallinity, and the processing conditions [32,48]. While its mechanical properties are suitable for applications requiring ductility, the softness of the materials and low modulus have motivated the use of PCL in blends or composites to improve structural performance. From a degradation standpoint, PCL undergoes slow hydrolytic degradation of its ester linkages, followed by microbial action, especially under composting or enzymatically active conditions [39]. PCL degrades quickly, within months, under conditions such as warm temperature (~30–40 °C), active microbial community, moisture, and slightly acidic conditions (pH ~5–7). However, it takes years to degrade under conditions such as limited microbial activity and low temperature. High crystallinity and hydrophobicity of PCL also slow the degradation rate [6,49]. While this slow degradation rate is beneficial for long-term biomedical uses such as scaffolds and drug delivery systems, it limits the effectiveness of PCL in short-lifespan packaging or agricultural applications unless it is blended with polymers with fast biodegradation kinetics or reinforced with hydrophilic fillers. The literature also highlights the limited polarity and hydrophobic nature of PCL, which often leads to poor compatibility with polar fillers and polymers. This has driven a considerable amount of research into surface modification, grafting, and compatibilization strategies to enhance its interfacial interactions in composites. Recent efforts include the functionalization of PCL chains and incorporation of nanofillers, such as NC, to improve both the mechanical and thermal performance of PCL-based materials while retaining biodegradability [23].

## 3. Synthesis and Properties of Poly(Lactic Acid)

**Figure 2:**
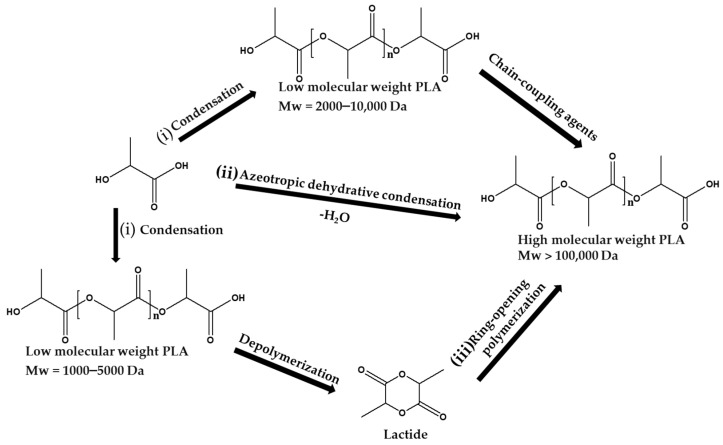
Different synthetic methods for the production of PLA (Adapted from [54], Open Access).

Poly(lactic acid) (PLA) is one of the most extensively studied biodegradable aliphatic polyesters due to its bio-based origin, good mechanical strength, and biocompatibility. According to Farah et al. [50] and Luyt et al. [51], PLA is primarily synthesized via (i) direct polycondensation of lactic acid, (ii) azeotropic dehydrative condensation, and (iii)  ROP of lactide, as illustrated in Figure 2. The ROP method is more favorable industrially due to the ability to achieve high molecular weight polymers and control over stereochemistry [51]. The synthesis process is typically catalyzed by tin(II) octoate, which allows for tuning of the molecular architecture of the polymer [52,53].

In general, PLA consists of crystalline domains formed by thin plate-like stacks of folded polymer chains, connected to amorphous zones by chain folds and tie molecules [55]. However, its physicochemical properties are closely linked to its stereochemistry. Research highlights that poly(L-lactic acid) is semi-crystalline with the degree of crystallinity of ca. 20–40% and exhibits a higher melting point (~170–180 °C), while poly(D,L-lactic acid) is mostly amorphous with the degree of crystallinity of ca. 0–10% and is more ductile [53]. These differences influence mechanical and thermal performance, with poly(L-lactic acid) showing higher tensile strength and modulus, whereas poly(D,L-lactic acid) offers better flexibility. The mechanical properties of PLA are well-established. Woodruff et al. [35] reported tensile strengths in the range of 50–70 MPa. However, the inherent brittleness and low impact resistance of PLA are frequently cited drawbacks, prompting research into plasticization, copolymerization, and blending with more ductile polymers such as PCL or poly(butylene adipate–*co*–terephthalate) (PBAT). PLA is biodegradable, primarily via hydrolysis followed by microbial action. However, it is worth noting that biodegradation rates are significantly influenced by crystallinity, molecular weight, and environmental conditions. While industrial composting can achieve efficient biodegradation, PLA tends to persist under ambient conditions, raising concerns about its suitability in certain applications without additional modifications [53,56].

## 4. Nanocellulose: Extraction Methods and Characterization Techniques

**Figure 3:**
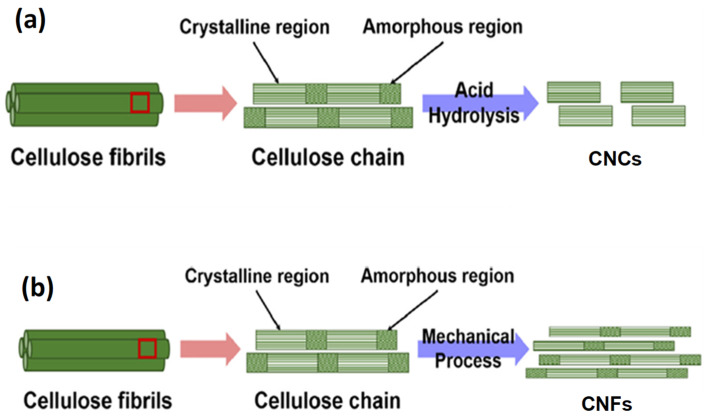
Schematic of the extraction of (**a**) CNCs and (**b**) CNFs from cellulose fibers via acid hydrolysis and mechanical process, respectively. The right-hand side represents a magnified view of the boxed region on the left to show cellulose at nanoscale (Adapted from [64], Open Access).

Nanocellulose (NC) is a renewable and biodegradable nanomaterial typically derived from lignocellulosic biomass [57]. It has emerged as a promising reinforcing agent in biopolymer composites due to its high mechanical strength, low density, and ease of surface modification. Studies in the literature have identified three main types of NC, namely cellulose nanocrystals (CNCs), cellulose nanofibrils (CNFs), and bacterial nanocellulose (BNC), each with distinct structural and physical characteristics [21,32,36]. Extraction typically begins with chemical pre-treatments of the starting biomass, such as alkaline delignification and bleaching to remove lignin and hemicellulose, yielding purified cellulose suitable for nanoscale processing. Figure 3 provides extraction methods, highlighting how they lead to different types of NC, namely CNCs (Figure 3a) and CNFs (Figure 3b). Each method has distinct effects on the properties of the resulting NC, which can further influence the performance of the final bio-nanocomposites. CNCs (Figure 3a) are mainly obtained via acid hydrolysis, with sulfuric acid being the most commonly used reagent. It breaks the amorphous cellulose regions and crystallizes the cellulose into highly ordered crystalline structures. However, this method can compromise thermal stability, prompting interest in alternative acids or enzymatic hydrolysis to mitigate such effects. CNFs (Figure 3b), on the other hand, are extracted through mechanical fibrillation methods including high-pressure homogenization and grinding, often aided by chemical or enzymatic pre-treatments such as 2,2,6,6-tetramethylpiperidine-1-oxyl (TEMPO)-mediated oxidation to enhance fibrillation efficiency and reduce energy demand [58,59,60]. In contrast, BNC is biosynthesized by microbial fermentation and is valued for its ultrafine network structure, high purity, and superior water-holding capacity, which make it especially suitable for biomedical applications [32]. The characterization of NC has been reported in the literature, with various studies highlighting how morphological, structural, and thermal properties influence their performance in polymer nanocomposites [58,59,61]. Transmission electron microscopy (TEM) and scanning electron microscopy (SEM) are frequently used to evaluate NC dimensions and morphology. According to the literature, sulfuric acid-derived CNCs typically appear as rigid, rod-like particles with lengths ranging from 100 to 300 nm and diameters between 5 and 20 nm, while CNFs exhibit entangled fibrillar structures several microns in length. X-ray diffraction provides insights into crystalline and polymorphic forms. CNCs extracted via acid hydrolysis typically exhibit higher crystallinity indices (ca. 60–90%) due to removal of amorphous regions [62,63]. Such high crystallinity enhances stiffness and thermal resistance, making CNCs suitable for reinforcing semi-crystalline polymers such as PCL and PLA.

Fourier transform infrared spectroscopy (FTIR) is widely used to confirm chemical composition and surface functional groups. The literature reports characteristic cellulose bands at ~3340 cm^−1^ (O–H stretching), ~2900 cm^−1^ (C–H stretching), and ~1050 cm^−1^ (C–O–C stretching), with additional peaks in modified NC, indicating successful surface functionalization [23,60]. For instance, esterification with citric acid or silanization with organo-silanes leads to peak shifts or the appearance of new bands, reflecting changed interfacial behavior. Thermogravimetric analysis (TGA) has been used to evaluate thermal stability, showing that CNCs produced via sulfuric acid hydrolysis degrade at lower temperatures (~180–250 °C) due to the presence of sulfate ester groups [65]. Differential scanning calorimetry (DSC) is used to observe transitions such as glass transition temperature (*T_g_*) and to evaluate the influence of NC on the thermal behavior of polymer blends. The literature shows the importance of applying multiple complementary techniques to fully characterize NC and predict its behavior in specific composite applications. Without such a comprehensive understanding, optimizing its reinforcing potential in PLA, PCL, and PLA/PCL blend matrices remains a challenge.

## 5. Poly(ε-caprolactone)/nanocellulose Bio-Nanocomposites: Preparation and Property Enhancements

The incorporation of NC into PCL matrices has gained significant attention in recent years. This is mainly because of its potential to address the limitations of neat PCL, such as low stiffness and moderate thermal resistance. The literature highlights various preparation techniques for PCL/NC bio-nanocomposites, with melt blending and solution casting being the commonly used (see Table 2). Melt blending offers the advantage of scalability and solvent-free processing, although achieving uniform NC dispersion remains a challenge due to its hydrophilic nature and tendency to agglomerate within the hydrophobic PCL matrix [21,66]. On the other hand, solution/solvent casting, often using chloroform or dichloromethane as solvents, facilitates better initial dispersion but is limited by environmental and processing drawbacks [20,22,23,36,67,68]. Electrospinning has emerged as an effective technique for fabricating nanofiber-based PCL/CNCs scaffolds [69]. In these systems, CNCs were well-aligned within the fibers, contributing to anisotropic mechanical enhancement. For example, electrospun mats containing CNCs from cotton waste fiber demonstrated up to 50% tensile strength improvement at 1 wt.% CNCs loading. However, at concentrations beyond 4 wt.%, CNCs tend to agglomerate, adversely affecting elongation at break and reducing toughness. Therefore, an optimal loading of 2–3 wt.% is often recommended to balance strength and flexibility [69]. To overcome dispersion issues, research has shown that surface modification strategies such as acetylation, silanization, or surfactant treatment of NC can be useful. PCL-grafted CNCs, obtained via ring-opening polymerization of caprolactone monomer using the “grafting from” approach, have also been reported [70]. For example, Avella et al. [66] demonstrated that treated CNCs improved interfacial adhesion and dispersion within PCL, resulting in enhanced mechanical performance compared to unmodified fillers. In another study, grafting CNFs with PCL (CNFs-*g*-PCL) improved the dispersibility of CNFs in the PCL matrix, which led to improved mechanical properties. Melting temperatures of the composites also increased, which improved the thermal processability of PCL [71].

In terms of property enhancements, numerous studies confirm that the addition of NC significantly improves the mechanical performance of PCL. The tensile strength, Young’s modulus, and elongation at break have all shown measurable increases at low NC loadings (typically < 5 wt.%) [21,22,23,36,67]. These improvements are attributed to the high aspect ratio and rigidity of NC, which contribute to effective stress transfer at the filler–matrix interface when dispersion is well controlled. In one study, N’Gatta et al. [72] designed 3D-printed bioactive composite scaffolds for bone tissue engineering using fused deposition modeling, incorporating PCL, CNCs, and silver nanoparticles (AgNPs). The binary PCL/CNCs composite displayed significantly higher stiffness and ductility compared to the ternary PCL/CNCs/AgNps system, highlighting CNCs’ role in enhancing the mechanical performance of the composite scaffolds. In another study, Chanthavong et al. [73] fabricated green composites based on PCL and polyvinyl alcohol (PVA) blend reinforced with polyethylene oxide (PEO)-modified cellulose microfibers. Interestingly, the incorporation of just 1 wt.% cellulose into the PCL–PVA blend significantly increased the tensile strength. The observed enhancement in tensile strength underscores the reinforcing ability of cellulose on the mechanical properties of these composite materials. Thermal behavior has also been positively affected; TGA and DSC analyses from multiple studies indicate that NC can enhance the thermal stability and crystallinity of PCL composites, although this effect is strongly dependent on the type of NC and its dispersion quality [22,66,67,68]. Moreover, NC acts as a nucleating agent, promoting faster crystallization kinetics, which is particularly advantageous for applications requiring rapid processing or dimensional stability [22]. From a biodegradation point, incorporation of hydrophilic NC into PCL increases the hydrophilic character of PCL/NC composites, potentially enhancing water penetration, hydrolysis of ester bonds, and access for microbial/enzymatic attack. Factors such as dispersion of NC on the PCL matrix and the reduced crystal size of NC contribute to increasing the degradation rate. Poor dispersion reduces water uptake, which hinders biodegradation. If the addition of NC significantly increases the crystallinity of the composite, the biodegradation rate decreases since the crystalline domains are less accessible to microbes and water compared to amorphous regions [74,75]. To summarize, the literature shows that NC is an effective, bio-based reinforcement for PCL. It offers multifunctional improvements that broaden the applicability of PCL in biomedical, packaging and environmental contexts, provided that interfacial compatibility and dispersion are properly managed.

**Table 2:** Summary of poly(ε-caprolactone)/nanocellulose bio-nanocomposites with source, structure, and application insights.

Composite	Source	Filler (wt.%)	Fabrication Method	Enhanced Properties	Application Area	References
PCL/CNCs	Sugarcane bagasse	2.5, 5.0, 7.5, 10.0, 12.5, and 15.0	Solution casting	Tensile modulus, storage modulus, biodegradability properties, and moisture barrier. Maximum increase in Young’s modulus was about 77%, and it was achieved at 12.5 wt.% CNCs loading.	Food packaging	[36]
PCL/CNCs	Commercial	10	Melt blending and pressing	Rheological performance, crosslinking, and thermal stability.	Shape-memory applications	[66]
PCL/CNCs	Marine algae biomass	0.5, 1.0, and 2.0	Solvent casting	Thermal stability, Young’s modulus, and tensile strength.	Packaging	[67]
PCL/CNCs	Purchased	1.0, 3.0, 5.0, and 7.0	Solvent casting	Nucleation and crystallinity for all composites.	Energy storage	[68]
PCL/CNCs	Sugarcane bagasse	2.0	Solution casting	Heterogeneous nucleation, crystallization rate, and improved tensile strength of PCL from 16.5 MPa to 17.8 MPa.	Packaging	[22]
PCL/CNCs	Orange peel	2.0	Solution casting	Heterogeneous nucleation, crystallization rate, and improved tensile strength of PCL.	Packaging	[22]
PCL/micro-cellulose	Wheat bran	2.0	Solution casting	Heterogeneous nucleation, crystallization rate, and improved tensile strength of PCL.	Packaging	[22]
PCL/CNCs	Starch	1.0, 3.0, and 5.0	Solution casting	Tearing strength increased by 68%, and oxygen transmission rate (from 1740 to 1250 cm^3^/m^2^ per day) and gas permeability were improved for the composite with 1.0 wt.% CNCs.	Packaging membrane	[23]
PCL-CaAlg/CNCs	Cotton	1.0 and 5.0	Solution casting	Degradation rate and hydrophilicity.	Wound dressing	[20]
PCL/CNFs	Rice straw	0.5, 1.0, 5.0, 10.0, and 15.0	Melt blending and pressing	Tensile strength and Young’s modulus increment of 7.5% at 10 wt.% CNFs and 76% at 15 wt.% CNFs, respectively. Hydrophilicity at 15 wt.% CNFs. Nucleated crystallization.	Packaging and biomedical	[21]
PCL/CNCs	Cotton waste fiber	0.5, 1.0, 1.5, 2.5, and 4.0	Electrospinning	Hydrophilicity and crystallization activation energy at 1.0 wt.% CNCs content.	Tissue engineering and wound dressing	[69]
PCL/CNFs-*g*-PCL	Hardwood kraft pulp	10.0, 20.0, and 30.0	3D printing	Young’s modulus and rigidity improved for all samples. Melting temperature increased. Tensile strength enhanced by 45.5% at CNFs-*g*-PCL 30.0 wt.% loading.	Sustainable products	[71]
PCL/CNCs	Purchased	1.0, 2.0, and 3.0 wt.%	Wet spinning	Balanced tensile strength and flexibility (Young’s modulus of 0.27 GPa). Biocompatibility.	Tissue engineering and anterior cruciate ligament	[76]

Note: PCL = polycaprolactone; CNCs = cellulose nanocrystals; CNFs = Cellulose nanofibrils; wt.% = weight percentage.

## 6. Poly(Lactic Acid)/nanocellulose Bio-Nanocomposites: Preparation and Property Enhancements

The integration of NC into PLA has been extensively studied as a strategy to overcome the inherent drawbacks of PLA, such as brittleness, low impact strength, and slow crystallization rate. The literature presents a variety of preparation methods for PLA/NC bio-nanocomposites, such as solvent casting, injection molding, melt blending, electrospinning, and extrusion. Solvent casting, often used in early-stage studies, enables good initial dispersion of NC in PLA due to solvent-assisted mixing [15,17,37,65,77]. However, industrial relevance has driven growing interest in melt processing techniques, which are environmentally favorable and scalable [14,18,19,78]. Despite its advantages, melt processing poses challenges related to the incompatibility between hydrophobic PLA and hydrophilic NC, often leading to agglomeration. Relative to PLA, unmodified NC has a higher surface energy owing to its polar nature at ambient conditions. A direct consequence of the surface energy difference is poor compatibility and thus weak interfacial adhesion between the two components, leading to a non-uniform distribution of the nanofiller in the polyester matrix. Surface modification of NC has been shown to lower its surface energy, improving the polymer-filler interaction and thus achieving uniform dispersion [79]. To address this, researchers have investigated surface modification techniques, such as grafting with glycidyl methacrylate, to enhance interfacial adhesion and dispersion of NC in PLA [18]. Grafting of PLA chains onto NC surfaces results in more homogeneous composites and superior mechanical behavior [80]. For example, incorporation of poly(ethylene glycol) (PEG)-grafted CNCs into PLA through electrospinning enhanced mechanical properties such as tensile strength and elongation at break. Properties such as thermal stability, hydrophilicity, and enzymatic degradation rate were also enhanced by CNCs-*g*-PEG incorporation [81]. Incorporation of CNFs-*g*-PLA to PLA through 3D printing improved thermal stability and mechanical properties such as Young’s modulus and tensile strength [71]. Significant mechanical property enhancements have been reported with the addition of NC to PLA (Table 3). As highlighted in the literature [14,15,16,17,18,19], tensile strength and Young’s modulus of PLA composites increased notably with the incorporation of well-dispersed CNCs or CNFs, even at low filler contents (<5 wt.%). These enhancements are largely due to the high stiffness and aspect ratio of NC, which enable efficient stress transfer across the matrix–filler interface. However, a trade-off in elongation at break is often observed, attributed to restricted polymer chain mobility, unless plasticizers or compatibilizers are introduced. Beyond mechanical improvements, thermal stability and crystallization behavior of PLA are positively influenced by NC [82]. Studies using DSC and TGA show that NC can act as a nucleating agent, accelerating PLA crystallization and enhancing its thermal resistance [14,16,17,65]. These effects are especially pronounced with CNCs due to their high surface area and crystalline nature.

From the biodegradation point, well-dispersed hydrophilic CNCs on the PLA matrix improve water uptake and increase the accessibility of ester bonds to microbes and hydrolysis, which speeds up the degradation rate [83]. Biodegradation is slowed by poor dispersion of CNCs on the PLA matrix or strong CNCs–PLA interfacial adhesion, which lowers water penetration and polymer chain mobility [84]. For example, Manzano et al. reported that the incorporation of CNCs alone into PLA decreases the degradation rate of the PLA/CNCs composite by 20%, but incorporating CNCs with micro-fibrillated cellulose into PLA increased the biodegradation of the composite by 60% under composting conditions. This might be due to the increased crystallinity of PLA by the high crystalline CNCs, which hinders microbial access and reduces water uptake. On the other hand, the inclusion of micro-fibrillated cellulose improved water uptake [85]. The literature confirms that NC is a highly effective, renewable nanofiller for PLA. It enables the development of biodegradable composites with tailored mechanical and thermal properties for use in biomedical, packaging, and environmental applications.

**Table 3:** Summary of poly(lactic acid)/nanocellulose bio-nanocomposites with source, structure, and application insights.

Composite	Source	Filler (wt.%)	Fabrication Method	Enhanced Properties	Application Area	References
PLA/CNCs	Sugarcane bagasse fiber	10.0	Solvent casting	Acid hydrolysis of cellulose was performed by sulfuric acid (S–CNCs) and phosphoric acid (P–CNCs). Both P–CNCs and S–CNCs improved thermal stability of PLA. TGA results had shown that PLA/P–CNCs exhibited higher thermal stability than PLA/S–CNCs nanocomposites.	Packaging	[65]
PLA/CNFs	Sugarcane bagasse	1.0, 2.0, 3.0, 4.0, and 5.0	Injection molding	Improvement in water resistance, thermal stability, and mechanical properties such as tensile and flexural strength, impact resistance, and fracture toughness was observed in nanocomposites with 2 wt.% CNFs loading.	Sustainable products	[16]
PLA/CNCs	Sugarcane bagasse	5.0, 10.0, and 15.0	Solvent casting	Improved thermal stability and tensile strength at 10.0 wt.%	Packaging	[17]
PLA/CNCs	Softwood pulp	1.0, 2.0, 3.0, and 5.0	Solution casting and co-extrusion	Improved storage moduli at 3.0 wt.%.	Packaging, Medical	[77]
PLA/CNCs	Purchased	1.0, 3.0, and 5.0	Melt blending	Improved tensile properties at 3.0 wt.%.	Packaging	[18]
PLA/CNCs	Neptune grass	1.0 and 3.0	Solvent casting	Accelerated degradation at 3.0 wt.%.	Food packaging	[37]
PLA/CNCs	Wood pulp	0.5	Melt blending and injection molding	Tensile strength increased from 57.9 to 79.6 MPa. Crystallinity increased from 35.9 to 42.5%.	Packaging	[19]
PLA/CNCs	Purchased	0.75, 1.0, and 2.0	Single screw extrusion	Thermal stability increased at 2.0 wt.% CNCs, and tensile strength increased by 18.2% at 1.0 wt.% CNCs.	3D biomedical applications.	[14]
PLA/CNCs	Purchased	2.0	Solvent-free cast extrusion	Reduced microbial growth, therefore, increased the shelf life of food that is oxygen sensitive.	Food packaging	[78]
PLA/CNCs-PEG	Cotton	0.5, 1.0, 2.0 and 4.0, and 8.0	Electrospinning	Tensile strength and elongation at break improved by factor of 2.8 and 1.9, respectively, at 4.0 wt.% CNCs-PEG loading, while thermal stability increased with increasing CNCs-PEG content. Hydrophilicity and enzyme degradation rate increased for all CNCs-PEG-containing samples compared to neat PLA.	Sustainable products	[81]
PLA/CNFs-g-PLA	Hardwood kraft pulp	10.0, 20.0, and 30.0	3D printing	Young’s modulus significantly increased at 20.0 wt.% CNFs-*g*-PLA loading. Tensile strength increased by 20.8% at 20.0 wt.% CNFs-*g*-PLA loading. Thermal stability is also enhanced by 20.0 wt.% CNFs-*g*-PLA loading.	Sustainable products	[71]
PLA/MCNCs	Purchased	1.25	Solution casting	Enhanced crystallinity, biodegradation rate under soil burial, and mechanical properties (tensile strength by 34.6% and elongation at break by 84.3%).	Agriculture and packaging	[86]
PLA/CNCs and PLA/CNCs-*g*-ECO	Purchased	1.0	Melt blending and (solvent casting followed by melt blending)	CNCs-*g*-ECO improved dispersion, acted as a nucleating agent, increased crystallinity, and thermal stability.	Sustainable products	[87]

Note: PLA = poly(lactic acid); CNCs = cellulose nanocrystals; MCNCs = Mineralized cellulose nanocrystals; PEG = poly(ethylene) glycol; ECO = Epoxidized canola oil TGA = Thermogravimetric analysis; wt.% = weight percentage.

## 7. Poly(Lactic Acid)/poly(ε-caprolactone) Biopolymer Blends: Morphology, Compatibility and Properties

**Figure 4:**
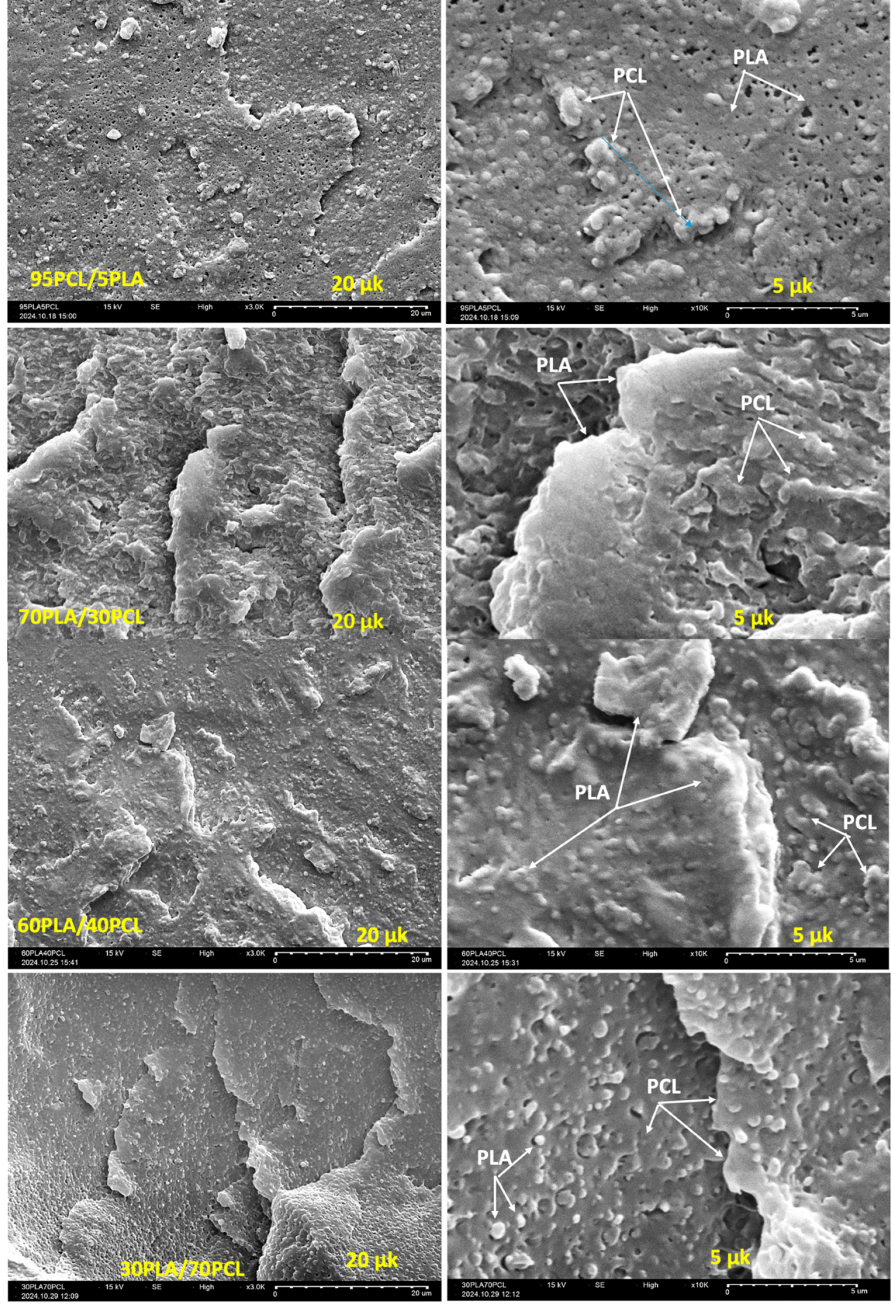
SEM micrographs of PLA/PCL blends at 95/5, 70/30, 60/40, and 30/70 *w*/*w* blend ratios at low (20 µm) and high (5 µm) magnifications. Arrows guide the reader to the PLA and the PCL phases. (Reproduced from [93], Open Access).

PLA and PCL blends have attracted considerable interest in the literature to synergistically combine the desirable properties of both polymers, thereby overcoming individual limitations. Table 4 summarizes the key findings from studies on PLA/PCL blends prepared mainly via melt blending, which remains the most widely used method due to its industrial relevance and scalability. PLA, characterized by high stiffness and brittleness, and PCL, known for its flexibility and toughness, are inherently immiscible due to differences in polarity and crystallinity, which results in phase-separated morphologies [26,27,88,89]. Morphological studies using SEM and TEM consistently reveal a dispersed droplet-matrix or co-continuous phase morphology, depending on the blend composition and processing conditions [88]. The degree of miscibility dictates the extent of molecular-level interactions between the polyesters, which in turn strongly influences the development of the blend morphology, and thus the mechanical properties and thermal behavior of the blends [90,91]. In one study, incorporating 10 wt.% of PCL in the blends improved the crystallinity of PLA, and the highest crystallinity was achieved at 20 wt.%, owing to the nucleating effect of PCL, which promotes the crystal formation of PLA. Interestingly, the crystallinity was significantly decreased with the utilization of higher PCL content (i.e., > 20 wt.%). This was attributed to the disruption of the PLA crystal structure by excess PCL, which hinders the effective chain arrangement [92]. In contrast, Ivanov et al. [93] reported that an increase in PLA crystallinity was not observed until the PCL content in the blends reached ca. 40 wt.%. The relatively low crystallinity of PLA at lower PCL loadings was attributed to the characteristic sea–island morphology observed in these immiscible blend systems (Figure 4). The study further demonstrated that the optimum size of the PCL (i.e., dispersed phase) was strongly dependent on the crystallinity of the PLA (i.e., continuous phase). Indeed, the low PLA crystallinity resulted in the formation of larger PCL spheres (≈1 µm), whereas higher PCL crystallinity promoted finer phase dispersion, leading to smaller PLA droplets (≈0.5 µm) in the 30PLA/70PCL blend (Figure 4). In other studies, this PLA/PCL blend ratio (i.e., 80/20) was also used to fabricate the blends and it was found that both the mechanical and thermal properties of the resulting 3D printed scaffolds were significantly improved [94,95]. Vala et al. [90] reported the preparation of PLA/PCL blends using PCL derivatives, where PCL was chemically modified with either maleic anhydride or glycidyl methacrylate. It was shown that he functionalization of PCL enhanced interfacial adhesion and consequently led to improved thermal stability of the blends. In general, at low PCL content, PCL droplets are dispersed within the PLA matrix, which typically leads to improved impact resistance but limited enhancement in ductility. At higher PCL loadings (>30 wt.%), co-continuous morphologies can form (Figure 4), resulting in balanced stiffness and toughness [89]. The major challenge reported in the literature is the limited compatibility between PLA and PCL. This is evident in blends with 30/70 and 80/20 PLA/PCL ratios, which exhibit uneven dispersion of PLA phases [26,88]. This leads to weak interfacial adhesion and poor stress transfer, resulting in compromised mechanical performance [26,88]. To address these compatibility challenges and further enhance the mechanical, thermal and functional properties of PLA/PCL blends, recent research has focused on reinforcing these systems with NC, ushering in a new generation of bio-nanocomposites with improved interfacial interactions and sustainability performance.

Property-wise, PLA/PCL blends exhibit a unique balance of mechanical, thermal, and barrier characteristics that can be tailored by adjusting the blend ratio and compatibilization approach. The literature reports that the addition of PCL generally improves the elongation at break and impact strength of PLA, mitigating its brittleness, while the stiffness and tensile strength tend to decrease proportionally with increasing PCL content [89]. Thermal analysis via DSC shows that blending can modify the crystallization behavior of both polymers, sometimes resulting in separate melting peaks indicative of phase immiscibility, although compatibilized blends can exhibit enhanced crystallinity due to improved phase interactions [27]. Furthermore, PLA/PCL blends maintain biodegradability and have been investigated for applications in packaging and biomedical devices [27,89].

## 8. Poly(Lactic Acid)/poly(ε-caprolactone)-Based Composites Hybrid Reinforcements: Processing and Performance

The development of PLA/PCL-based composites reinforced with fillers, such as NC and other bio- or inorganic reinforcements, has emerged as a promising approach to simultaneously enhance mechanical and thermal properties beyond what is achievable with single fillers. The literature shows that reinforcement strategies improve interfacial adhesion, dispersion, and composite functionality [24,27,31,32,33,34,89]. Processing methods for these nanocomposites typically involve melt blending, solvent casting, extrusion, injection molding, and 3D printing, with melt blending being the most industrially viable. However, achieving uniform dispersion and strong interfacial bonding remains a major challenge [96], often addressed through surface modification of fillers or compatibilizers tailored to interact with both the polymer matrix and the various fillers [25,32]. Performance-wise, studies summarized in Table 5 report that composites of PLA/PCL exhibit improved tensile strength, modulus, and toughness compared to neat PLA/PCL blends [24,25,31,32]. For example, the PLA/PCL/TEMPO-oxidized bacterial cellulose (TOBC) composite, manufactured via 3D printing and using TEMPO oxidation surface modification, showed a 17.4% increase in tensile strength, alongside a 208% increase in elongation at break. This indicates improved ductility and strength, making it well-suited for biomedical applications where flexibility and resilience are important. The enhanced crystallinity (~60%), associated with TOBC, also supported better structural integrity [32]. In contrast, the PLA/PCL/microcrystalline cellulose (MCC) composites demonstrated improved hydrophilicity and accelerated biodegradation, valuable attributes for a sustainable packaging solution. The melt extrusion and blending fabrication technique ensures effective dispersion of MCC at 1.0–3.0 wt.%, contributing to a moderate crystallinity (~45%) that balances mechanical performance and environmental degradability [33]. Interestingly, MCC composites did not report additional surface modification, suggesting a natural compatibility between MCC and the PLA/PCL matrix. Incorporation of 1.0 wt.% CNCs, as well as CNCs-*g*-PCL and CNCs-*g*-PLLA nanofillers, into the PLA/PCL matrix via melt-blending-accelerated biodegradation, improved compatibility, which enhanced mechanical properties such as elastic modulus and tensile strength [97]. CNCs, PCL-PEG-PCL tri-block copolymer (BCP), and BCP-CNCs were incorporated into the PLA/PCL (60/40) matrix for phase compatibility improvement. BCP10-CNCs1.0 enhanced interfacial interaction, 10.0 wt.% BCP enhanced the crystallinity of PCL, and the porosity increased with CNCs content in the blend [28].

The “optimal” NC content largely depends on the polyester (i.e., PLA or PCL), blend ratio (for PLA/PCL blends), morphology, and chemical modification of NC, the presence or absence of a compatibilizer, processing method, as well as target properties. For example, in one study, 3 wt.% NC was found to be optimal for dispersion and overall properties in PLA [98]; however, another study reported an optimal NC content of 4 wt.% in PCL [5]. Although the optimal values are system specific, relatively low nanofiller contents (<5 wt.%) lead to substantial enhancements in mechanical properties. Since NC has a high surface area, low concentrations permit even distribution within the matrix, promoting effective stress transfer. However, at high loadings, the filler particles aggregate, owing to strong intermolecular hydrogen bonding. These agglomerates no longer reinforce, but rather act as stress concentrators, initiating cracks and thus weakening the mechanical properties of the composites. Concerning the plastic deformation mechanism, PLA, being glassy at ambient conditions, is dominated by crazing and crack propagation. In contrast, PCL shows ductile behavior, allowing shear yielding and necking. However, in PLA/PCL blends, the deformation mechanism largely depends on interfacial adhesion, blend composition, and temperature [99]. Blending PLA with PCL, as well as the incorporation of CNCs into either matrix, can significantly influence the phase composition of these polyesters. For instance, CNCs have been shown to enhance the crystallinity of PCL [21,22,68,69]. Similarly, it has been reported to accelerate the crystallization kinetics of PLA, thereby increasing its overall crystallinity [19,86,87]. These observations clearly highlight the role of CNCs as an effective nucleating agent within these matrices, promoting a more ordered and less amorphous polymer structure. Nevertheless, this nucleating effect is not exclusive to CNCs; as the dispersion of PCL in PLA (continuous phase) has also been found to facilitate faster crystallization kinetics, while the incorporation of PLA into PCL (continuous phase) similarly contributes to an increase in crystallinity [93].

Concerning the mechanism of biodegradation, warm and moist conditions are essential, and hydrolysis is central to, and thus the initiating and rate-determining step in the process. Both PCL and PLA are aliphatic polyesters, comprising ester linkages along their backbones. It therefore logically follows that they are both susceptible to hydrolytic cleavage, albeit to varying degrees, depending on factors such as crystallinity, molecular weight, and hydrophobicity. Relative to NC, which is rich in hydroxyl groups, both PCL and PLA are hydrophobic; therefore, incorporation of cellulose nanofiller in either polyester’s matrix reduces the lipophilic-to-hydrophilic ratio of the resulting composite material, enhancing the water uptake and thus promoting hydrolysis. In the case of blends, the PCL-PLA interface is typically a region of structural discontinuity, owing to the immiscibility between the two polyesters. In addition, NC has been shown to often localize in these regions and compatibilizes the two components. Therefore, given the fibrous and porous nature of cellulose, these weak interfacial areas increase water diffusion into the blend composites and accessibility for microbial attack.

Nanoclay fillers such as montmorillonite (MMT) and organo-modified MMT (O.MMT) enhance the mechanical performance of the composites primarily by improving the interface interaction between the PLA and PCL phases. Surface functionalization of MMT nanoclay promotes better polymer compatibility and increases tensile strength, essential for applications like printing plates [25]. The O.MMT variant notably doubles the indentation modulus compared to neat PCL, indicating a significant increase in stiffness, which is desirable for rigid packaging materials [34]. These composites were typically prepared via melt blending, a method that effectively distributes nanoclay particles within the polymer matrix. Synthetic additives such as Pluronic and triallyl isocyanurate (TAIC) serve as compatibilizers that enhance mechanical properties by improving the miscibility of PLA and PCL. TAIC, added via melt blending, not only increases strength and modulus but also reduces phase separation in PLA/PCL blends, expanding its use in both packaging and biomedical fields [31]. Pluronic, incorporated at 2.5 to 7.5 parts per hundred and processed through melt blending, improves tensile strength at optimal blend ratios, targeting packaging applications [24]. Additionally, the inclusion of natural biopolymer reinforcements such as SFNPs improves the thermal stability and compatibility of PLA/PCL composites without the need for surface modifications. Melt blending these SFNPs at 1.0 wt.% offers benefits suitable for food packaging, where thermal resistance and safety are priorities [27]. Meanwhile, higher loading of natural fibers, such as flax fiber, at 20 wt.%, significantly enhances mechanical properties through extrusion and injection molding, demonstrating their potential for robust packaging materials [24]. In general, this diverse range of fillers and fabrication methods highlights the adaptability of PLA/PCL composites. NC and natural fibers contribute to environmental sustainability and biodegradability, while nanoclays and synthetic compatibilizers address mechanical performance challenges related to phase incompatibility. The choice of surface modification, filler loading, and processing technique is crucial in tuning the final properties for targeted applications, whether in packaging, biomedical devices, food packaging, or printing technologies.

**Table 5:** Overview of poly(lactic acid)/poly(ε-caprolactone)-based composites with various fillers.

Composite Name	PLA/PCL Ratios	Filler Type and Source	Filler Content (wt.%)	Fabrication Method	Enhanced Properties	Application Area	References
PLA/PCL/TOBC	100/0, 95/5, 90/10, 85/15, and 80/20	TEMPO-oxidized bacterial cellulose	1.5	3D printing	10% PCL content increased tensile strength and elongation at break by 17.4% and 208% compared to that of neat PLA, respectively. Crystallinity increases with increasing PCL content.	Biomedical	[32]
PLA/PCL/MCC	90/10 and 80/20	Micro-crystalline cellulose (MCC) from cotton	1.0	Melt extrusion and blending	Enhanced hydrophilicity and accelerated biodegradation.	Packaging	[33]
PLA/PCL/CNCs	70/30	CNCs, CNCs-*g*-PCL and CNCs-*g*-PLLA	1.0	Melt blending	Enhanced shape-memory response, accelerated biodegradation, elastic modulus, and tensile strength.	Biomedicine and food packaging	[97]
PLA/PCL/CNCs, PLA/PCL/BCP, and PLA/PCL/BCP-CNCs	60/40	Cellulose nanocrystals (CNCs) from cotton and PCL-PEG-PCL (BCP) tri-block copolymer	CNCs (0.5, 1.0 and 2.0) BCP (5.0, 10.0 and 20.0)	Solvent casting	Enhanced water uptake for all samples. BCP10-CNCs1.0 enhanced interfacial interaction. 10.0 wt.% BCP enhanced crystallinity of PCL. Porosity increased with CNCs content in blend.	Biomedical	[28]
PLA/PCL/MMT nanoclay	80/20	Montmorillonite (MMT)	2.0, 4.0, and 6.0	Melt blending	Higher tensile strength and compatibility at 4.0 wt.% MMT.	Printing plates	[25]
PLA/PCL/Silk fibroin nanoparticles	100/0, 90/10, 80/20, and 70/30	Silk fibroin nanoparticles (SFNPs) from silkworm cocoons	1.0	Melt blending	Enhanced thermal stability and compatibility for 70/30 blend by 1.0 wt.% SFNPs.	Food packaging	[27]
PLA/PCL/ Pluronic	100/0, 90/10, 85/15, 80/20, 75/25, and 70/30	Synthetic Pluronic	2.5, 5.0, and 7.5	Melt blending	Improved tensile strength for blends with 10, 15, and 20 wt.% PCL content at 2.5 parts per hundred.	Packaging	[24]
PLA/PCL/TAIC	80/20, 60/40, 40/60, and 20/80	Triallyl isocyanurate (TAIC)	3.0	Melt blending	Improved strength, modulus, and hindered phase separation for 20 PLA/80 PCL.	Packaging and Biomedical	[31]
PLA/PCL/MMT	70/30	Montmorillonite	1.0	Solvent casting, Melt blending	Improved PLA phase dispersion and better interface interaction.	Biomedical	[26]
PLA/PCL/ O.MMT	20/80	Organophilic Montmorillonite (O.MMT)	2.0	Melt blending	Improved indentation modulus by 50% compared to that of PCL.	Packaging	[34]
PLA/PCL/CNCs	70/30	CNCs (purchased)	1.0, 2.0, 3.0, and 5.0	Melt extrusion and blending	Improved compatibility and mechanical properties.	Various applications	[100]

Note: CNCs = cellulose nanocrystals; MCC = Microcrystalline cellulose; O.MMT = Organo-modified montmorillonite; PCL = Poly(ε-caprolactone); PLA = Poly(lactic acid); SFNPs = Silk fibroin nanoparticles; TAIC = Triallyl isocyanurate; TEMPO = 2,2,6,6-tetramethylpiperidine-1-oxyl; TOBC = (TEMPO)-oxidized bacterial cellulose.

## 9. Conclusions, Challenges, and Future Perspectives

The incorporation of nanocellulose (NC) into biodegradable polymers such as PLA and PCL has gained considerable attention in overcoming the inherent limitations of PLA and PCL. This includes the brittleness of PLA and the weak thermal and mechanical stability of PCL. This study focuses on the structure–property correlations and performance factors of PLA/NC, PCL/NC, PLA/PCL, and PLA/PCL/NC systems. The addition of NC functions as a reinforcing and nucleating agent, increasing stiffness, modulus, and, in some circumstances, biodegradation rate, depending on dispersion and surface functioning. Surface modification of NC (e.g., acetylation, silanization, or grafting with compatibilizers such as PLA or PCL chains) was found to be the most efficient in improving interfacial adhesion and dispersion inside hydrophobic matrices. For PLA/PCL composites, compatibilization with reactive agents and optimal blend ratios (usually 70/30 or 60/40 PLA/PCL) were discovered to be crucial for balancing stiffness, ductility, and biodegradability. The addition of NC to the PLA/PCL matrix increased mechanical reinforcement, crystallinity control, and interphase stability, provided that good dispersion was achieved. Key parameters influencing overall blend performance include NC surface chemistry, dispersion uniformity, blend content, degree of crystallinity, and processing technique and conditions. A synergistic balance between hydrophilic NC and the immiscible PLA/PCL phases can be achieved through appropriate compatibilization or surface functionalization, resulting in better structural integrity and adaptable degradation profiles. The cost of NC production, energy-intensive extraction and modification processes, and dispersion issues in polymer matrices continue to be barriers to large-scale implementation from an industrial and economic standpoint. However, the increasing demand for bio-based, compostable materials, as well as advancements in reactive extrusion and continuous NC manufacturing technologies, make these systems more and more viable for packaging, biomedical scaffolds, and agricultural films. Overall, the integration of NC into PLA, PCL, and their blends provides a viable pathway toward high-performance, biodegradable composites.

Despite the promising potential of bio-nanocomposites, several challenges must be addressed to facilitate their widespread adoption and further advancement. These challenges include processing, interface compatibility, environmental assessments, and application enhancements. One significant challenge is based on optimizing the processing techniques for bio-nanocomposites to ensure consistent material properties and performance. Factors such as physical, chemical, and mechanical characteristics of the constituent materials must be carefully considered to prevent variations that could render the nanocomposite unsuitable for its intended use. Interface compatibility between polymer components, particularly in blends such as PLA and PCL, presents another hurdle. Immiscibility and weak interface interactions can limit the overall performance of the composite, necessitating the development of effective compatibilizers to enhance toughness and ensure homogeneity. Another challenge is industrial scalability, NCs’ high cost and restricted availability, mostly due to energy-intensive extraction and purification, continue to be major challenges to commercialization. NC production costs typically vary from ZAR 173.16 to ZAR 346.32 per kg, whereas commodity fillers like CaCO_3_ cost less than ZAR 34.63 per kg. Furthermore, establishing uniform dispersion of NCs inside hydrophobic polymer matrices necessitates surface modification or solvent-assisted compounding, which increases processing steps and costs. Regulatory aspects include adhering to food-contact requirements in packaging applications. PLA is already certified for food packaging, but NCs must adhere to purity and migration standards, particularly if made from plant sources containing residual lignin or hemicellulose. In biomedical applications, both PLA and PCL are approved by the Food and Drug Administration for specific implantable and drug-delivery applications; nevertheless, NCs must demonstrate biocompatibility and cytotoxicity before being integrated into medical devices. Comprehensive life cycle assessments (LCAs) are essential for evaluating the overall environmental impact of bio-nanocomposites. Assessments should cover the entire life cycle from component extraction to final disposal. This will ensure that bio-nanocomposites contribute positively to environmental sustainability and economic efficiency. Enhancing the properties and applications of bio-nanocomposites requires ongoing research and innovation.

Despite higher raw material costs, CNCs-reinforced PLA and PCL composites can reduce environmental impact through renewable sourcing and faster biodegradation, enabling compliance with extended producer responsibility and biodegradability certification frameworks. As NC production becomes more cost-effective, industrial adoption is expected to advance in specialized high-value industries such as biomedical scaffolds or biodegradable barrier films before spreading to commodity sectors. Future research should further explore the design, processing, and characterization of NC-reinforced PLA/PCL blend nanocomposites to improve breakage resistance, hydrophilicity, and water barrier properties in food packaging applications, as well as enhance cell attachment and drug release kinetics in biomedical applications. Key areas of focus should include optimizing dispersion methods, tailoring surface chemistry for interfacial compatibility, and understanding the synergistic effects of ternary systems on biodegradation, crystallinity, and functional performance. Exploring new applications in electronic devices, energy production, and environmental remediation will further expand the scope of bio-nanocomposites and contribute to sustainable technologies. Addressing the aforementioned challenges and advancing this line of investigation holds substantial promise for developing high-performance, scalable, and truly sustainable materials for packaging, agriculture, biomedical devices, and other environmentally critical sectors.

## Figures and Tables

**Table 1:** Comparison of properties of poly(ε-caprolactone) and poly(lactic acid) [35,36,37,38].

Polymer	Tensile Strength (MPa)	Elongation at Break (%)	Melting Temperature (°C)	Glass Transition (°C)	Processing Method	Approximate Degradation Time Under Composting Conditions (Months)	Applications
PCL	10–40	200–600	58–64	−65 to −60	Extrusion, injection molding, compression molding, solvent casting, electrospinning	>24	Drug delivery sutures
PLA	50–70	3–6	173–178	60–65	Extrusion, injection molding, compression molding, solvent casting	6 to 12	Orthopedic surgery, oral and maxillofacial surgery

**Table 4:** Processing methods and performance outcomes of poly(lactic acid)/poly(ε-caprolactone) blends.

Blend Composition (PLA/PCL wt.%)	Fabrication Technique	Key Processing Characteristics	Findings/Impact on Composite Performance	Applications	References
30/70	Melt blending	High temperature, direct mixing	Incompatible blends; poor interface adhesion; uneven PLA phase dispersion.	Not reported	[26]
100/0, 90/10, 80/20, 70/30, 60/40, 50/50, 40/60, 30/70, 20/80, 10/90, and 0/100	Melt blending	High temperature, direct mixing	Stiffness, strength, elongation at break, thermal stability, and activation energy balancing were enhanced for (60/40) PLA/PCL blend. However, other blends showed poor compatibility and mechanical properties.	Packaging, Biomedical	[89]
80/20	Melt blending	High temperature, direct mixing	Large, dispersed particle sizes; poor mechanical properties.	Not reported	[88]
100/0, 90/10, 80/20, 70/30, and 0/100	Melt blending	High temperature, direct mixing	All blends showed poor compatibility. Based on results, blend containing 30% PCL had superior thermal properties compared to other blend ratios.	Packaging, Biomedical	[27]

Note: PLA = Poly(lactic acid); PCL = Poly(ε-caprolactone).

## Data Availability

No new data were created or analyzed in this study. Data sharing is not applicable to this article.

## Abbreviations

The following abbreviations are used in this manuscript:

AgNPs	Silver nanoparticles
CNCs	Cellulose nanocrystals
CNFs	Cellulose nanofibers
DSC	Differential scanning calorimetry
FTIR	Fourier transform infrared spectroscopy
MCC	Microcrystalline cellulose
MMT	Montmorillonite
NC	Nanocellulose
O.MMT	Organically modified montmorillonite
PBAT	Poly(butylene adipate–*co*–terephthalate)
PCL	Poly(ε-caprolactone)
P–CNCs	Phosphoric acid cellulose nanocrystals
PEG	Poly(ethylene) glycol
PEO	Poly(ethylene oxide)
PVA	Poly(vinyl alcohol)
PLA	Poly(lactic acid)
ROP	Ring-opening polymerization
rROP	Radical ring-opening polymerization
S–CNCs	Sulfuric acid cellulose nanocrystals
SEM	Scanning electron microscopy
SFNPs	Silk fibroin nanoparticles
TAIC	Triallyl isocyanurate
TEM	Transmission electron microscopy
TEMPO	2,2,6,6-Tetramethylpiperidine-1-oxyl
TGA	Thermogravimetric analysis
TOBC	(TEMPO)-oxidized bacterial cellulose
